# Sex Differences in Circadian Clock Genes and Myocardial Infarction Susceptibility

**DOI:** 10.3390/jcdd8050053

**Published:** 2021-05-08

**Authors:** Ivana Škrlec, Jasminka Talapko, Martina Juzbašić, Robert Steiner

**Affiliations:** 1 Faculty of Dental Medicine and Health, Josip Juraj Strossmayer University of Osijek, HR-31000 Osijek, Croatia; jtalapko@fdmz.hr (J.T.); mjuzbasic@fdmz.hr (M.J.); 2 Faculty of Medicine, Josip Juraj Strossmayer University of Osijek, Josipa Huttlera 4, HR-31000 Osijek, Croatia; steiner_robert5@hotmail.com; 3 Clinical Department of Cardiovascular Diseases and Intensive Care, Clinic for Internal Medicine, University Hospital Osijek, Josipa Huttlera 4, HR-31000 Osijek, Croatia

**Keywords:** biological sex, circadian rhythm, clock genes, myocardial infarction

## Abstract

The growing body of evidence shows a significant difference in the circadian rhythm of cardiovascular disease based on biological sex. The incidence of cardiovascular disease varies between women and men. Additionally, biological sex is vital for the timely application of therapy—chronotherapy, which benefits both sexes. This study aimed to examine the potential difference of single nucleotide polymorphisms (SNPs) of the circadian rhythm genes *ARNTL*, *CLOCK*, *CRY2* and *PER2* in women and men with myocardial infarction. A cross-sectional study was conducted, including 200 patients with myocardial infarction. Altogether, ten single nucleotide polymorphisms in the *ARNTL*, *CLOCK*, *CRY2* and *PER2* genes were analyzed. The Chi-square test yielded statistically significant differences in *CLOCK* gene rs11932595 polymorphism in a recessive genotype model between women and men with a p-value of 0.03 and an odds ratio 2.66, and a corresponding 95% confidence interval of 1.07 to 6.66. Other analyzed polymorphisms of the circadian rhythm genes *ARNTL*, *CRY2*, and *PER2* did not significantly differ between the sexes. According to the study’s current results, the *CLOCK* gene’s genetic variability might affect myocardial infarction concerning biological sex.

## 1. Introduction

Circadian rhythm affects daily the physiological functioning that is important for the proper functioning of peripheral organs, including the heart [1]. The central molecular clock is located in the suprachiasmatic nucleus (SCN) and controls peripheral molecular clocks located in almost all organs [2]. Thus, circadian rhythms are essential for normal physiology, and if there is a desynchronization between the central and peripheral clocks, disease develops [3,4]. The molecular clock’s basis consists of a transcriptional and translational feedback loop of four key transcription factors. CLOCK and ARNTL (BMAL1) heterodimers form a positive loop that stimulates *PER* and *CRY* transcription and other clock-linked genes. A negative feedback loop is composed of PER and CRY heterodimers, which inhibits the transcription of *CLOCK* and *ARNTL* genes [5,6].

Circadian rhythm influences daily body functions such as temperature, hormone release, digestion, sleep, and mood [7,8]. Many cardiovascular functions are also regulated by circadian rhythms, such as blood pressure, heart rate, endothelial function, and thrombus formation [3,9,10,11,12]. Circadian rhythm disorders can have negative consequences for the whole organism, including the cardiovascular system, which leads to an increased risk of heart disease [13,14].

Numerous studies have shown a significant difference in circadian rhythm on cardiovascular disease (CVD) based on sex [3]. It has been shown that women are somewhat more sensitive to circadian rhythm disorders and have an increased risk of chronic diseases affecting the reproductive and immune systems, central nervous system, endocrine functions, and cardiovascular health [7,8]. Many physiological processes that are regulated by circadian rhythms differ between women and men. Therefore, women have higher systolic and diastolic blood pressure at night than men [15]. Women have a higher heart rate [16], are more sensitive to sleep disorders than men [8], and have been shown to have a shorter circadian period than men [17]. Many CVDs are associated with obesity, the onset of which is mainly sex-related [18]. Sex plays a vital role in the circadian disorder of energy homeostasis [19]. Women are more sensitive to insulin and have higher adiponectin and leptin levels, while men have higher triglycerides levels in the blood [18,19,20]. Circadian disorder in women stimulates adipose tissue storage, while fat stores’ mobilization is more effective in men [18]. Men are less susceptible to circadian rhythm disorders [19]. Different triggers could affect the various incidence of CVD between men and women. These triggers may be due to exposure to multiple factors, sex-specific cardiac mechanisms, or both [21].

The circadian rhythm influences heart disease in a sexually dimorphic manner [9]. Women and men differ in their resistance to cardiovascular disease, with sex hormones and sex chromosomes playing an important role [3,9,18,22,23,24]. Studies in rodents have shown that female mice have a higher survival rate after myocardial infarction (MI) than male mice [25]. Additionally, despite disturbed circadian rhythm, female mice are protected from the development of metabolic changes and cardiomyopathy [13]. Sex hormones, estrogens, are thought to play a protective role against CVD development, including MI. Estrogens influence circadian molecular clock gene expression, which is essential in regulating circadian rhythm via SCN [22]. Premenopausal women have less CVD than men of the same age, but that changes when women reach menopause. Then, estrogen’s cardioprotective effect is lost, and elevated testosterone levels lead to hypertension and coronary artery disease in postmenopausal women [24,26]. Cardiolipin composition was observed to differ in female and male mice’s heart cells, which may affect MI resistance in females [9]. Cardiolipins are an essential part of mitochondria’s inner membrane and play a crucial role in mitochondria’s bioenergetics, and their composition is altered in heart disease [13]. Another possible explanation for the lower incidence of CVD in women is that women may have unrecognized CVD symptoms, including myocardial infarction, and are less likely to report chest pain associated with acute coronary syndrome [27]. Additionally, it is known that a large number of women with acute MI die before hospitalization [27]. Studies have shown a significant reduction in morbidity and mortality from atherosclerosis in men, while the rate of recurrent atherothrombotic events, including cardiovascular death, has increased in women [28]. The mechanisms of circadian rhythm act differently in the hearts of women compared to men. Women and men show flexibility in cardiovascular disease, and biological sex is crucial for circadian therapy to benefit both sexes [9].

All of the mentioned above led to this study’s aim to investigate the potential difference in *ARNTL*, *CLOCK*, *CRY2*, and *PER2* circadian rhythm gene polymorphisms in women and men with myocardial infarction.

## 2. Participants and Methods

### 2.1. Participants

**Figure 1:**
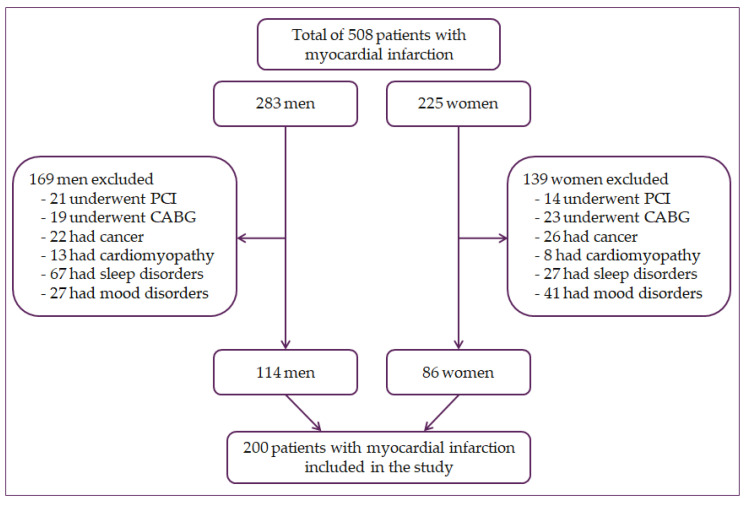
Flowchart of patient selection. PCI—percutaneous coronary intervention, CABG—coronary artery bypass grafting.

A cross-sectional study was conducted, including 200 patients with myocardial infarction. Of these, 86 were women, and 114 were men. Subjects were patients with a history of myocardial infarction hospitalized at the University Hospital Osijek, Croatia. Patients with type I and II myocardial infarction with elevated cardiac troponin T above the 99th percentile were included in the study and with one of the subsequent factors: electrocardiogram (ECG) changes, pathological Q waves, myocardial ischemia symptoms, loss of viable myocardium or regional wall motion abnormalities, or coronary thrombus identified by angiography [29,30]. Patients were excluded from this study if they had undergone percutaneous coronary intervention (*n* = 35) or a coronary artery bypass (*n* = 42) because those are not clinical characteristics of acute MI. Additionally, patients were excluded if they had cancer (*n* = 48), cardiomyopathy (*n* = 21), or other circadian rhythm-regulated diseases, such as sleep disorders (*n* = 94) and mood disorders (*n* = 68), including depression (Figure 1).

Data on the patient’s medical history and sociodemographic data were collected. All data were additionally checked in the patient’s medical records.

The Ethics Committee of the University Hospital Osijek (Ethical Approval Code: 25-1: 3160-3/2012) approved this research. According to the Declaration of Helsinki and its amendments, the study was carried out. All participants signed informed consent forms.

### 2.2. Genotyping of Single Nucleotide Polymorphisms

In this study, SNPs were determined in four circadian rhythm genes: *CLOCK*, *ARNTL*, *CRY2*, and *PER2*. A total of ten SNPs were analyzed. Three SNPs in the *ARNTL* gene (rs3789327, rs4757144 and rs12363415), and *CLOCK* (rs11932595, rs6811520 and rs13124436). Meanwhile, two SNPs were analyzed in the *CRY2* (rs2292912 and rs10838524) and *PER2* genes (rs35333999 and rs934945). These central clock gene polymorphisms have been associated with cardiovascular risk factors in earlier research [14,31,32,33]. All tested polymorphisms are located in the intronic region of the genes, except for *PER2* gene polymorphisms.

According to the manufacturer’s standard protocol, the extraction of genomic DNA was made from peripheral blood lymphocytes (QIAamp DNA Blood Mini Kit, Qiagen, Hilden, Germany). All analyses were conducted by real-time PCR using TaqMan probes. Allele discrimination analyses were performed with SDS 7500 software version 2.3 (Applied Biosystems, Foster City, CA, USA).

### 2.3. Statistical Analyses

Statistical analyses were carried out with SPSS software (version 22.0, SPSS Inc., Chicago, IL, USA) for Windows. Quantitative data are shown as the mean and standard deviation, while categorical data are shown as frequency and percentages. For each SNP, a significant difference was calculated to compare allele frequency and genotype distribution in women and men using the Chi-Square test (χ2) on contingency tables. An additional level of genotyping quality control was achieved by applying the Chi-square goodness-of-fit test and analyzing genotypes’ distribution with those expected in the Hardy–Weinberg equilibrium. Genotype models were determined using the web tool SNPStats [34]. Logistic regression was used to estimate the effect of *ARNTL* (rs3789327, rs4757144, rs12363415), *CLOCK* (rs11932595, rs6811520, rs13124436), *CRY2* (rs2292912 and rs10838524) and *PER2* (rs35333999 and rs934945) genotypes on the patients’ sex. Age, smoking, history of hypertension, dyslipidemia, type 2 diabetes mellitus, positive family history of CVD, history of former CVD, systolic and diastolic blood pressure, and BMI were used as a covariate. Kruskal–Wallis and Chi-square tests were utilized to define the association of cardiovascular risk factors with polymorphisms of the analyzed molecular clock genes. In the case where *p* ≤ 0.05, the association was considered significant. Significant value corrections were made by the Benjamini–Hochberg method (false detection rate—FDR value) due to several polymorphisms tested. Only q-values less than 0.05 were considered significant. The most common haplotypes and the association between haplotypes and CVD risk factors were identified by the SNPStats web tool [34]. Only an additive model was considered due to the low frequency of some haplotypes. 

## 3. Results

Demographic and clinical characteristics of women and men are presented in Table 1. The mean age of all participants was 66 ± 12 years, and 57% were men. 

After Bonferroni correction, a significant difference was found in the recessive genotype model of *CLOCK* rs11932595 polymorphism (GG vs. AG + AA) in women compared to men (*p* = 0.03, the odds ratio of 2.66, 95% confidence interval 1.07 to 6.66). Other genotype models of *ARNTL*, *CRY2* and *PER2* genes were not significantly different between women and men (Table 2).

Logistic regression was adjusted to evaluate the independent impact of the chosen polymorphism after modifying for age and cardiovascular risk factors. All tested SNPs showed a significant interaction between age (*p* = 0.017), smoking (*p* < 0.001), and diastolic blood pressure (*p* = 0.045) (Table 3).

The chi-square test compared the allelic frequency and genotype distribution between women and men (Table 4). Women and men were not significantly different in tested polymorphisms or genotype distribution in *ARNTL*, *CLOCK*, *CRY2*, or *PER2* genes.

The association was found among cardiovascular risk factors and examined circadian rhythm gene polymorphisms in women and men (Table 5). The SNP rs12363415 in the *ARNTL* gene was associated with type 2 diabetes mellitus in women (*p* = 0.003). In contrast, rs2292912 in the *CRY2* gene and rs934945 in the *PER2* gene were associated with dyslipidemia in the men (*p* = 0.02, and <0.001, respectively). The *CLOCK* polymorphism rs11932595 was associated with systolic and diastolic blood pressure in women (*p* = 0.005 and *p* = 0.006, respectively) and BMI in men (*p* = 0.024).

The haplotypes were examined in the four circadian clock genes. The frequencies of the predicted haplotypes of the tested circadian clock gene polymorphisms in women and men are shown in the supplementary material (Supplemental Table S1). The haplotype CGA distribution at the *ARNTL* gene locus was statistically significantly different when comparing haplotype frequency between women and men (*p* = 0.030, OR = 1.86, 95% CI = 1.05–3.27). The CGA haplotype of the *ARNTL* gene was significantly associated with dyslipidemia and hypertension under the additive model (*p* = 0.03, OR = 0.45, 95% CI = 0.22-0.95, and *p* = 0.03, OR = 0.29, 95% CI = 0.09–0.88, respectively). Furthermore, a significant difference was observed at the *CRY2* gene locus when comparing the haplotype GG frequency between women and men (*p* < 0.001, OR = 9.73, 95% CI = 9.71–9.76). The CA and GA haplotypes of the *CRY2* gene were significantly associated with dyslipidemia under the additive model (*p* = 0.02, OR = 8.20, 95% CI = 1.42–47.45, and *p* = 0.03, OR = 6.44, 95% CI = 1.09–37.89, respectively).

## 4. Discussion

The genetic variability in the *ARNTL*, *CLOCK*, CRY2 and *PER2* genes in this study showed that variations in the circadian genes differ between women and men with myocardial infarction. The present study found evidence of the difference between women and men in the recessive genotype model of the rs11932595 in the *CLOCK* gene in the sample of 200 patients with myocardial infarction. Circadian rhythm plays a vital role in cardiac aging, which differs between men and women [9]. That is why biological sex is essential in the right approach to studying circadian rhythm in cardiovascular diseases [9].

In this study, women had a higher prevalence of dyslipidemia. Moreover, the dyslipidemia prevalence was significantly higher in women than in men. Additionally, women were significantly older than men which is similar to in the previous study [35]. Women with CVD are usually older and have more risk factors than men. Thus, both hyperlipidemia and hypertension are more common in women with MI than in men [35], which is evident in this study’s results. Some of the tested circadian rhythm genes were associated with some of the CVD risk factors in women and men with MI. Thus, *ARNTL* rs12363415 was connected with type 2 diabetes mellitus in women, while *CRY2* rs2292912 and *PER2* rs934945 were connected with dyslipidemia in men. The *CLOCK* rs11932595 was associated with systolic and diastolic blood pressure in women and BMI in men.

Circadian rhythm disorders in humans are associated with cardiometabolic diseases [23]. Circadian rhythm is influenced by sex, and this interaction is evident throughout most of life [23]. Women are more resistant than men to circadian rhythm disorders due to sleep disorders and shift work [23]. Circadian rhythm disorders due to shift work or sleep disorders are associated with an increased risk of cardiovascular disease, especially in men [13]. Studies in rodents have found significant sex differences in heart size and function, and sex influences cardiovascular aging [36]. Additionally, mutations in several central clock genes have been linked to many cardiovascular diseases [36].

Mutations in the *CLOCK* gene lead to age- and sex-dependent dilated cardiomyopathy. Aging female mice do not develop cardiomyopathy, even when the circadian rhythm is disrupted due to *CLOCK* gene mutation [9]. However, *ARNTL* (*BMAL1*) knock-out male mice show premature aging [9]. In mice with a *CLOCK* gene mutation, a circadian period is prolonged. Female mice remain healthy for a long time and do not develop cardiac dysfunction, unlike male mice that develop cardiac hypertrophy and dysfunction quickly [23]. This effect is lost after ovariectomy. In this case, the cardiometabolic function is impaired in female mice [23]. Female biological sex and estrogens lessen heart disease development and have a cardioprotective effect [13].

The lower prevalence of the rs11932595 polymorphism in women (8.1% versus 18.4% in men) could mean two things. First, more women with the GG genotype die before surviving myocardial infarction and enrolling in the present study. Second, fewer women with the GG genotype suffered a myocardial infarction and were hospitalized. Thus, one cannot infer whether this is beneficial or detrimental. Nevertheless, patients carrying the *CLOCK* rs11932595 GG genotype were 2.66 times likely to have myocardial infarction. 

Genetic variations in the *CLOCK* gene are connected with blood pressure and cardiovascular disorders in humans [37,38,39,40]. Here, an association of rs11932595, located in the CLOCK gene’s intronic region, with systolic and diastolic blood pressure was found in women and with BMI in men. *CLOCK* knock-out and *CLOCK* mutant mice show an essential role of CLOCK in regulating blood pressure and kidney function [38,39,40]. Mice with a knock-out or mutated CLOCK gene have lower blood pressure, plasma aldosterone levels, changed urinary sodium excretion, and a higher incidence of diabetes insipidus than control mice [37,38,39,40]. A possible mechanism contributing to this is the reduced level of 20-hydroxyeicosatetraenoic acid in the *CLOCK* knock-out mice’s urine [37,39,40]. The 20-hydroxyeicosatetraenoic acid is essential in regulating blood pressure and blood flow [37,39,40]. The CLOCK protein has also been associated with diabetes and obesity due to a mutation that prevents CLOCK from binding to DNA and activating target genes. Overweight women with a specific polymorphism in the 3′ untranslated region of the *CLOCK* gene have decreased gene expression activity, altered sleep patterns, and circadian rhythmicity [39]. Studies conducted on *CLOCK* mutant mice have shown the importance of CLOCK protein in daily pulse rate control, myocardial contractility, and basal metabolism [41]. Mutation of the *CLOCK* gene in cardiomyocytes affects only the circadian rhythm in cardiomyocytes and leads to physiological changes in cardiomyocytes. Some of these changes are cardiac metabolism, heart rate, contractility, response to external signals, cardiac growth, and regeneration [42].

In addition to *CLOCK* gene mutations that have been shown to affect sex-related CVD development, research has shown that mutations in the *PER1* gene also affect blood pressure differently in men and women [25]. There is no dipping in blood pressure overnight in *PER1* knock-out male mice [25]. Such males develop non-dipping hypertension and have an increased risk of developing CVD. *PER1* knock-out female mice have a steady drop in blood pressure overnight and are protected from hypertension without dipping [25]. Sex hormones contribute to sex differences in blood pressure and cardiovascular parameters [25].

Some studies have shown that haplotypes of the *ARNTL* gene are associated with hypertension and type 2 diabetes [43,44]. In the present study, the frequency of the CGA haplotype of the *ARNTL* gene was 19% in women versus 11% in men, and patients carrying the mentioned haplotype were 0.45 and 0.29 times less likely to have dyslipidemia and hypertension, respectively. Kovanen et al. found the association of the *CRY2* haplotype with elevated triglycerides and metabolic syndrome [45]. This study’s haplotype analysis further supports this phenotype association of dyslipidemia with the *CRY2* polymorphisms and haplotypes. It is observed that dyslipidemia is associated with rs2292912 *CRY2* polymorphism in men, and patients carrying the CA or GA haplotypes of the *CRY2* gene were 8.2 and 6.44 times likely to have dyslipidemia, respectively.

Understanding biological sex differences in cardiovascular function regulation by circadian rhythm plays an essential role in applying drug therapy or chronotherapy [9,46]. Biological sex is a crucial factor in the timely administration of drugs in clinical cardiology [9]. There are sex differences in the incidence, pathophysiology, and clinical presentation of heart disease. Women with ischemic heart disease have worse outcomes and are less likely to receive timely treatment than men [47,48]. More women, after menopause, have dyslipidemia than men [47], which is seen from the results of this study. Additionally, that contributes to the higher incidence of CVD in women after menopause. Moreover, the same diagnostic thresholds for cardiac troponin in both sexes could not be used because women with myocardial infarction have a minor cardiac troponin increase. The sex-specific diagnostic limits could improve the diagnosis of myocardial infarction in women. In women with suspected ischemic heart disease, fewer tests are performed, and often the condition remains undiagnosed. That is why women have a worse prognosis in ischemic heart disease and die more often than men [47]. All of the above might be the reason why fewer postmenopausal women are included in the study.

This study had several limitations. Sex-related differences in cardiovascular disease disappear when women reach menopause. Since the average women’s age in this study was 69 years, we did not find otherwise present sex-related differences in the CVD. An additional limitation is the study sample size. A higher number of participants could yield a better association between circadian clock gene polymorphisms and myocardial infarction. Furthermore, probably, analyzed polymorphisms in the *ARNTL*, *CRY2*, and *PER2* genes are not functionally associated with myocardial infarction. In this case, functional SNPs or a set of functionally relevant polymorphisms that could be strongly associated with myocardial infarction should be named. This approach to the problem of myocardial infarction provides an opportunity to continue research in multicenter studies with many participants. This research’s advantage is that the participants involved were homogeneous in demographic background characteristics such as ethnicity.

## 5. Conclusions

This study showed that genetic variation of the *CLOCK* gene was a biological sex difference in myocardial infarction patients. Premenopausal women have a lower prevalence of cardiovascular disease than men of the same age due to estrogen’s protective effect. However, after menopause, the prevalence of diseases like coronary heart disease and heart failure increases dramatically and exceeds the prevalence in men of the same age. In this study, postmenopausal women were older than men. This age difference might contribute to a higher incidence of CVD in women with worse outcomes, which is a possible reason why no more significant differences were observed between men and women in the present study. The application of circadian biology in clinical practice represents a significant possibility of reducing patients’ morbidity or mortality after myocardial infarction. For both sexes to benefit from CVD treatment, biological sex should be considered for chronotherapy. Additional research and analysis of the sex-related circadian clock are possible.

## Figures and Tables

**Table 1:** Demographic and clinical characteristics of patients.

Variable	Women	Men	*p*-Value
Number (%)	86 (43%)	114 (57%)	-
Age (years)	69 ± 12	64 ± 12	**0.002** *
Smoking	16 (18.6%)	25 (21.9%)	**<0.001** †
Hypertension	50 (58.1%)	57 (50%)	0.25 †
Dyslipidemia	17 (19.8%)	9 (7.9)	**0.013** †
Type 2 diabetes mellitus	18 (20.9%)	26 (22.8%)	0.75 †
Positive family history of CVD	29 (25.4%)	18 (20.9%)	0.18 †
History of former CVD	82 (71.9%)	61 (70.9%)	0.33 †
Systolic blood pressure (mm Hg)	127.55 ± 18.21	126.14 ± 13.89	0.97
Diastolic blood pressure (mm Hg)	75.77 ± 10.86	77.27 ± 9.25	0.46
BMI (kg/m^2^)	29.31 ± 4.19	28.30 ± 5.07	0.09

* Mann–Whitney U test, † Chi-squared test, BMI—body mass index. The bold are statistically significant values.

**Table 2:** Genotype models of the *ARNTL*, *CLOCK*, *CRY2,* and *PER2* polymorphisms.

Gene	Codominant Model			Dominant Model			Recessive Model		
	*p*	OR	95% CI	*p*	OR	95% CI	*p*	OR	95% CI
*ARNTL*									
rs3789327	0.18	0.56	0.29–1.07	0.06	0.56	0.30–1.04	0.56	0.80	0.37–1.70
rs4757144	0.48	1.28	0.69–2.39	0.67	1.13	0.63–2.02	0.36	0.69	0.31–1.52
rs12363415	0.77	1.05	0.56–1.99	0.98	1.01	0.54–1.88	0.48	0.43	0.04–4.89
*CLOCK*									
rs11932595	0.08	0.93	0.50–1.72	0.67	1.14	0.63–2.05	**0.03**	2.66	1.07–6.66
rs6811520	0.45	0.78	0.42–1.46	0.28	0.72	0.40–1.31	0.33	0.68	0.31–1.47
rs13124436	0.95	0.97	0.53–1.77	0.99	1.00	0.57–1.77	0.76	1.15	0.46–2.87
*CRY2*									
rs2292912	0.99	0.97	0.54–1.76	0.94	0.98	0.55–1.75	0.96	1.05	0.17–6.60
rs10838524	0.30	1.46	0.75–1.97	0.46	1.26	0.68–2.36	0.28	0.67	0.33–1.38
*PER2*									
rs35333999	0.89	1.24	0.42–3.72	0.79	1.15	0.41–3.21	0.77	0.66	0.04–10.76
rs934945	0.43	1.23	0.64–2.34	0.79	1.09	0.59–2.01	0.25	0.39	0.07–2.11

*p*-Values shown in the table are corrected for the multiple comparisons. The bold is statistically significant value.

**Table 3:** Odds ratios for the difference between sex, adjusted for age, and cardiovascular risk factors included in the logistic regression model.

Risk Factor	OR (95% CI)	*p* Value
Age	0.96 (0.94–0.99)	**0.017**
Smoking	2.24 (1.45–3.47)	**<0.001**
Hypertension	1.06 (0.53–1.44)	0.86
Dyslipidemia	2.36 (0.85–6.56)	0.10
Type 2 diabetes mellitus	0.85 (0.37–1.95)	0.71
Positive family history of CVD	1.20 (0.69–2.09)	0.51
History of former CVD	0.92 (0.59–1.44)	0.72
Systolic blood pressure (mm Hg)	0.97 (0.94–1.00)	0.07
Diastolic blood pressure (mm Hg)	1.05 (1.01–1.11)	**0.045**
BMI (kg/m^2^)	0.96 (0.89–1.03)	0.27

BMI—body mass index. The bold are statistically significant values.

**Table 4:** Allele and genotype distribution and frequencies of the *ARNTL*, *CLOCK*, *CRY2*, and *PER2* polymorphisms.

Gene	SNP	Minor Allele	MAF * Women	MAF * Men	*p*-Value	*q* Value	Genotype	Genotype Frequency, N (%)				
								Women	Men	*p* Value	Χ^2^	*q* Value
*ARNTL*	rs3789327	C	0.46	0.38	0.118	0.125	TT	23 (26.7%)	44 (38.5%)	0.210	3.11	0.216
							TC	47 (54.6%)	53 (46.4%)			
							CC	16 (18.6%)	17 (14.9%)			
	rs4757144	G	0.38	0.37	0.823	0.835	AA	35 (40.6%)	44 (38.5%)	0.578	1.09	0.577
							AG	36 (41.8%)	55 (48.2%)			
							GG	15 (17.4%)	15 (13.1%)			
	rs12363415	G	0.16	0.15	0.828	0.888	AA	61 (70.9%)	81 (71%)	0.698	0.72	0.757
							AG	23 (26.7%)	32 (28%)			
							GG	2 (2.3%)	1 (0.8%)			
*CLOCK*	rs11932595	G	0.35	0.41	0.214	0.255	AA	32 (37.2%)	41 (35.9%)	0.103	4.53	0.102
							AG	47 (54.6%)	52 (45.6%)			
							GG	7 (8.1%)	21 (18.4%)			
	rs6811520	T	0.42	0.36	0.220	0.254	CC	29 (33.7%)	46 (40.3%)	0.465	1.53	0.486
							CT	41 (47.6%)	53 (46.4%)			
							TT	16 (18.6%)	15 (13.1%)			
	rs13124436	A	0.32	0.33	0.846	0.914	AA	9 (10.4%)	13 (11.4%)	0.976	0.05	1
							AG	37 (43%)	49 (42.9%)			
							GG	40 (46.5%)	52 (45.6%)			
*CRY2*	rs2292912	G	0.20	0.20	1	1	CC	53 (61.6%)	71 (62.2%)	0.982	0.03	0.999
							CG	31 (36%)	40 (35%)			
							GG	2 (2.3%)	3 (2.6%)			
	rs10838524	A	0.45	0.45	1	1	GG	27 (31.3%)	30 (26.3%)	0.327	2.23	0.317
							GA	40 (46.5%)	65 (57%)			
							AA	19 (22%)	19 (16.6%)			
*PER2*	rs35333999	T	0.05	0.05	1	1	CC	79 (91.8%)	104 (91.2%)	0.952	0.09	0.999
							CT	6 (6.9%)	9 (7.8%)			
							TT	1 (1.1%)	1 (0.8%)			
	rs934945	T	0.18	0.16	0.722	0.789	CC	60 (69.7%)	78 (68.4%)	0.241	2.84	0.247
							CT	21 (24.4%)	34 (29.8%)			
							TT	5 (5.8%)	2 (1.7%)			

* MAF—minor allele frequency, *q* value—corrected significant *p*-value by the Benjamini–Hochberg method.

**Table 5:** The association between cardiovascular risk factors and circadian clock gene SNPs.

Gene	Age *		Smoking †		Hypertension †		Dyslipidemia †		Type 2 Diabetes Mellitus †		Positive Family History of CVD †		History of Former CVD †		SBP *		DBP *		BMI *	
	Women	Men	Women	Men	Women	Men	Women	Men	Women	Men	Women	Men	Women	Men	Women	Men	Women	Men	Women	Men
*ARNTL*																				
rs3789327	0.425	0.391	**0.011**	0.895	0.118	0.406	0.233	0.314	0.182	0.339	0.181	0.088	0.071	**0.034**	0.330	0.209	0.348	0.475	0.607	0.183
rs4757144	0.264	0.111	0.718	0.182	0.187	0.383	0.199	0.477	0.642	0.184	0.057	0.172	**0.038**	0.527	0.696	0.729	0.297	0.164	0.106	0.609
rs12363415	0.634	0.587	0.737	0.595	0.170	0.566	0.094	0.875	**0.003**	0.848	0.152	0.992	0.122	0.443	0.931	0.831	0.318	0.927	0.523	0.774
*CLOCK*																				
rs11932595	0.190	0.567	0.426	0.937	0.269	0.617	0.173	0.060	0.798	0.224	0.276	0.702	0.693	0.724	**0.005**	0.157	**0.006**	0.275	0.996	**0.024**
rs6811520	0.439	0.169	0.304	0.691	0.687	0.560	0.399	0.431	0.378	0.693	0.389	0.463	0.313	0.501	0.499	0.327	0.592	0.161	0.334	0.219
rs13124436	0.891	0.561	0.651	0.924	0.215	0.527	0.772	0.514	0.980	0.168	0.355	0.849	0.443	0.241	0.895	0.856	0.869	0.983	0.191	0.546
*CRY2*																				
rs2292912	0.818	0.366	0.811	0.562	0.169	0.178	0.768	**0.020**	0.188	0.908	0.840	0.579	0.139	0.303	0.749	0.503	0.750	0.515	0.531	0.834
rs10838524	0.135	0.544	0.215	0.776	0.338	0.138	0.288	0.175	0.606	0.224	0.879	0.575	0.095	0.857	0.202	0.814	0.500	0.844	0.274	0.080
*PER2*																				
rs35333999	0.194	**0.035**	0.101	0.632	0.624	0.574	0.391	0.896	0.365	0.860	0.274	**0.029**	0.713	0.525	0.607	0.149	0.368	0.511	0.423	0.217
rs934945	0.457	0.096	0.529	0.107	0.146	**0.034**	0.499	**<0.001**	0.933	0.162	0.819	0.785	0.129	**0.004**	0.305	0.195	0.264	0.668	0.345	0.449

The table presents * Kruskal–Wallis test *p*-value for numerical data and † Chi–squared test *p*-value for categorical data. CVD—cardiovascular disease, SBP—systolic blood pressure, DBP—diastolic blood pressure, BMI—body mass index. The bold are statistically significant values.

## Data Availability

The dataset generated during the study is available from the corresponding author on reasonable request.

## Supplementary Materials

The following are available online at https://www.mdpi.com/article/10.3390/jcdd8050053/s1, Table S1: Frequencies and distribution of probable haplotypes in the women and the men.
