# Supplementary material for: Sex Differences in Circadian Clock Genes and Myocardial Infarction Susceptibility
Source: J Cardiovasc Dev Dis. 2021 May 8;8(5):53. doi: 10.3390/jcdd8050053 (PMC8151899; doi:10.3390/jcdd8050053)
Supplement: Supplementary file 1 [file jcdd-08-00053-s001.zip › jcdd-1140734-supplementary.pdf]

Supplementary Material

Table S1: Frequencies and distribution of probable haplotypes in the women and men.

Gene	rs3789327	rs4757144	rs12363415	Frequency women	Frequency men	p value
ARNTL	T	A	A	0.43	0.45	0.67
	C	A	A	0.17	0.14	0.72
	C	G	G	0.08	0.09	0.75
	C	G	A	0.19	0.11	**0.03**
	T	G	A	0.09	0.15	0.13
CLOCK	rs11932595	rs6811520	rs13124436			
CLOCK	A	T	G	0.40	0.34	0.26
	G	C	A	0.11	0.13	0.61
	G	C	G	0.22	0.26	0.33
	A	C	G	0.05	0.05	0.94
	A	C	A	0.19	0.19	0.99
CRY2	rs2292912	rs10838524				
CRY2	G	A		0.25	0.25	0.92
	C	A		0.20	0.19	0.88
	G	G		0.00	0.01	**<0.001**
PER2	rs35333999	rs934945				
PER2	C	C		0.77	0.78	0.81
	C	T		0.18	0.17	0.72
	T	C		0.05	0.05	0.94
